# Clinical implications of the quantitative detection of ID4 gene methylation in myelodysplastic syndrome

**DOI:** 10.1186/s40001-015-0092-x

**Published:** 2015-02-20

**Authors:** Huiyuan Kang, Xinrong Wang, Li Gao, Jian Cen, Mianyang Li, Wei Wang, Nan Wang, Yonghui Li, Lili Wang, Li Yu

**Affiliations:** Department of Hematology, Chinese PLA General Hospital, 28 Fuxing Road, Beijing, 100853 China; Department of Clinical Tests, Chinese PLA General Hospital, 28 Fuxing Road, Beijing, 100853 China

**Keywords:** ID4 gene, DNA methylation, Myelodysplastic syndrome, Leukemia

## Abstract

**Background:**

Myelodysplastic syndrome (MDS) eventually transforms into acute leukemia (AL) in about 30% of patients. Hypermethylation of the inhibitor of DNA binding 4 (ID4) gene may play an important role in the initiation and development of MDS and AL. The aim of this study was to quantitatively assess ID4 gene methylation in MDS and to establish if it could be an effective method of evaluating MDS disease progression.

**Methods:**

We examined 142 bone marrow samples from MDS patients, healthy donors and MDS-AL patients using bisulfite sequencing PCR and quantitative real-time methylation-specific PCR. The ID4 methylation rates and levels were assessed.

**Results:**

ID4 methylation occurred in 27 patients (27/100). ID4 gene methylation was more frequent and at higher levels in patients with advanced disease stages and in high-risk subgroups according to WHO (*P* < 0.001, *P* < 0.001, respectively) and International Prognostic Scoring System (IPSS) (*P* = 0.002, *P* = 0.007, respectively) classifications. ID4 methylation levels changed during disease progression. Both methylation rates and methylation levels were significantly different between healthy donor, MDS patients and patients with MDS-AL (*P* < 0.001, *P* < 0.001, respectively). Multivariate analysis indicated that the level of ID4 methylation was an independent factor influencing overall survival. Patients with MDS showed decreased survival time with increased ID4 methylation levels (*P* = 0.011, hazard ratio (HR) = 2.371). Patients with ID4 methylation had shorter survival time than those without ID4 methylation (*P* = 0.008).

**Conclusions:**

Our findings suggest that ID4 gene methylation might be a new biomarker for MDS monitoring and the detection of minimal residual disease.

## Background

Myelodysplastic syndrome (MDS) is a heterogeneous group of clonal stem cell disorders characterized by abnormal differentiation and maturation of myeloid cells. The natural history of these syndromes may range from a chronic course, spanning many years, to a rapid course of leukemic progression [1], and 30% of cases of MDS eventually transform into acute leukemia (AL) [2]. In myelodysplasia, malignant transformation at the level of a myeloid stem cell can result in chromosomal abnormalities that act as signatures of the disease and can be identified in precursor cells giving rise to granulocytes, monocytes, red cells, and platelets [3]. For the development of the leukemic clone, further genetic events are required for the rapid expansion of leukemic blasts [4]. Many people regard MDS as the first stage of a hematological myeloid malignancy and consider MDS as the best intermediate stage research model for AL.

MDS pathogenesis and its frequent progression to AL are associated with aberrant cell clones with little ability for differentiation but with a high ability to proliferate [5,6]. This aberration of MDS is believed to be a multistep process of aberrant expression of the tumor suppressor gene (TSG) requiring both aberrant genetic and aberrant epigenetic factors such as abnormal hypermethylation of the gene promoter region [4,7-11]. Previous studies have demonstrated that abnormal hypermethylation of many TSG promoter regions such as CpG islands are associated with MDS development and progression [12-17]. MDS patients treated with hypomethylating agents such as 5-azacytidine and decitabine achieved high overall response rates and hematological improvement rates [3,18-22]. In addition, it is well known that with the development of MDS, there are increases in abnormal blasts in bone marrow, which may be the result of abnormal expression of TSG in terms of cell cycle, cell differentiation, and cell proliferation.

To date, the role of aberrant promoter methylation in MDS has not been investigated exhaustively, and previous studies have mainly focused on cycling-dependent kinase inhibitors even if other genes have been found to be targeted by aberrant hypermethylation in MDS. For example, hypermethylation of the inhibitor of DNA binding 4 (ID4) gene may play an important role in hematological [23] and solid cancers [24]. ID4 contributes to cell differentiation and proliferation [24-28], and abnormal methylation of the ID4 gene in hematological cancers plays an important role in diagnosis and prognosis [26,29]. Aberrant methylation of the ID4 promoter region is associated with invasiveness, growth, and recurrence of many solid tumors [26,29-34] and with initiation and progression in hematological cancers including leukemia and lymphoma [23,26,29,35]. However, the methylation status of the ID4 gene in MDS patients and its clinical significance are poorly described.

Due to the high frequency of DNA methylation in MDS and its involvement in leukemia transformation, we hypothesized that the analysis of DNA methylation levels is a potential predictor for prognosis. This was achieved using a rapid and quantitative methylation detection methodology based on real-time PCR. The aim of the present study was to investigate the clinical impact of ID4 gene methylation on MDS prognosis in terms of methylation rate and methylation levels of the ID4 gene.

## Methods

### Subjects for study

We selected 142 bone marrow samples harvested from the ilium by osteostixis from individuals who visited the Chinese PLA General Hospital between May 2007 and June 2010. Samples for bisulfite sequencing study corresponded to bone marrow specimens from a healthy donor (age: 45 years, gender: male), bone marrow from a patient with MDS who was diagnosed with refractory anemia (MDS-RA) (age: 40 years, gender: male) and bone marrow from a patient with acute myeloid leukemia (MDS-AML) (M6) (age: 42 years, gender: male). Both patients with MDS and AML were diagnosed according to the World Health Organization (WHO) criteria. The prognostic score for each patient was calculated using the International Prognostic Scoring System (IPSS) [36]. For methylight analysis, we selected bone marrow samples from 100 patients with MDS, 20 healthy donors and 22 patients with MDS-AML from our institution. Ten samples in each group were randomly selected to assess the correlation between bisulfite sequencing PCR (BSP) and methylight; methylight was performed only in patients with high BSP results because of the detection threshold of methylight. Patients’ characteristics are shown in Table 1. Normal bone marrow samples (*n* = 20) were obtained for negative controls. In addition, for further positive and negative controls, we included malignant hematological cell lines including NB4 (acute leukemia cell line) and 293 (renal cell line) obtained from the American type culture collection (ATCC).

**Table 1:** **Clinical characteristics of the study groups**

**Group**	**Number of patients (total** ***n*** **= 142)**
NBM	20
MDS-AL	22
MDS	100
WHO subtype	100
Low-risk	70
RA	41
RARS	7
RCMD	22
High-risk	30
RAEB-1	21
RAEB-2	9
IPSS risk group	35
Low-risk	24
Low	2
Int-1	22
High-risk	11
Int-2	11
High	0
Karyotype	
Good	22
Poor	6

NBM, normal bone marrow; MDS, myelodysplastic syndrome; AL, acute leukemia; RA, refractory anemia; RARS, refractory anemia with ringed sideroblasts; RCMD, refractory cytopenia with multilineage dysplasia; RCMD-RS, refractory cytopenia with multilineage dysplasia and ringed sideroblasts; RAEB, refractoryanemia with excess of blasts; IPSS, International Prognostic Scoring System.

The study was approved by the local internal review boards and ethics committees. Written informed consent was obtained from all subjects.

### DNA extraction

DNA was extracted using a Genomic DNA extraction kit (Promega, Madison, WI, USA). The ID4 gene DNA sequence was obtained from the National Center for Biotechnology Information (NCBI).

### Bisulfite sequencing PCR

Sodium bisulfite treatment was provided to 1 μg of each DNA sample using the Epi bisulfite kit (Qiagen, Venlo, Limburg, Netherlands) according to the manufacturer’s instructions. Bisulfite-treated genomic DNA was altered so that all unmethylated CpG sites were converted to TG, and methylated CpG sites remained CG.

**Figure 1:**
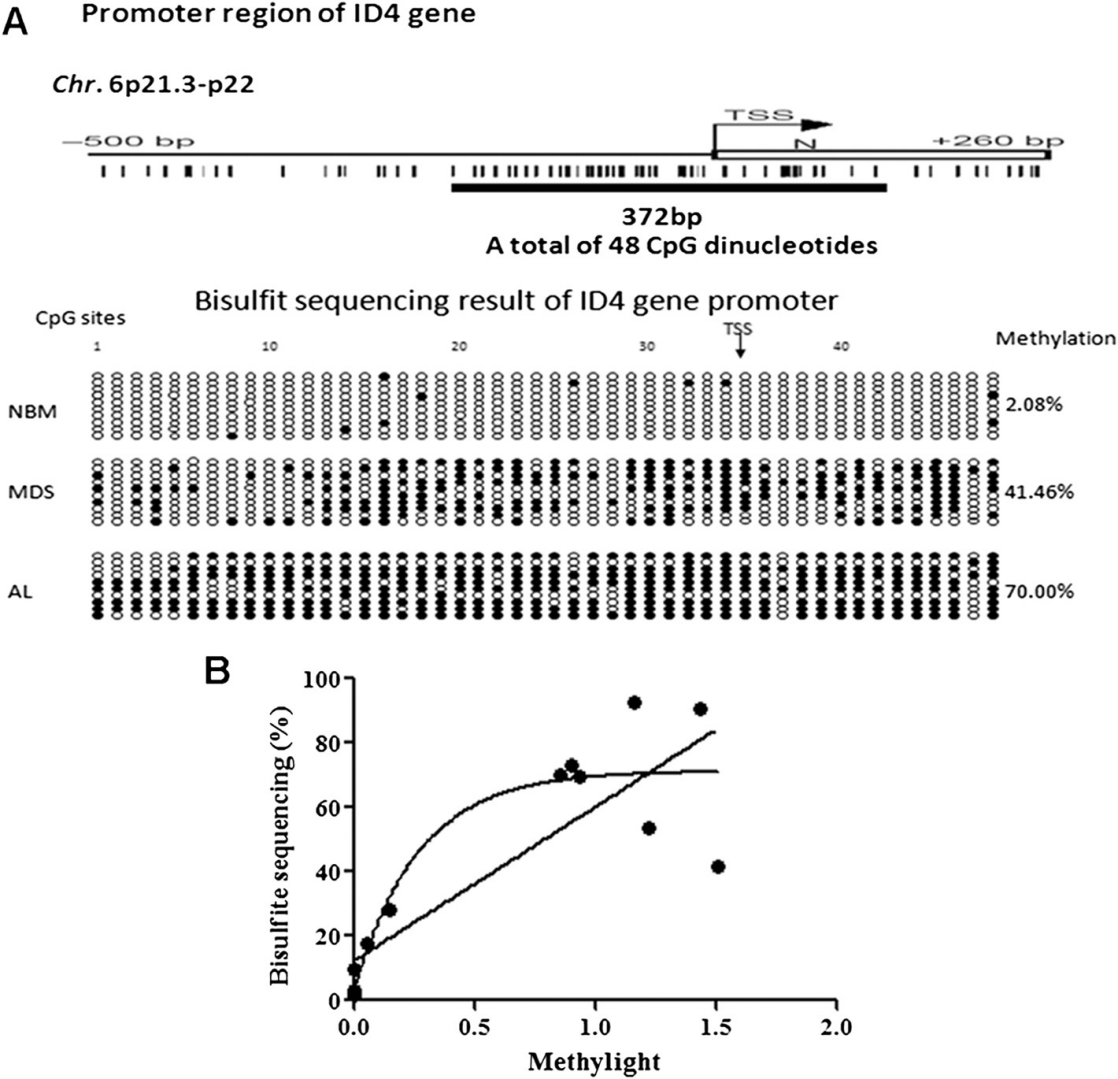
**Methylation frequency of CpG sites and association between the ID4 methylation percentage. (A)** Methylation frequency of CpG sites in the ID4 gene in NBM, MDS and MDS-AL. Filled circle and empty circle represent one methylated CpG site (CG) and unmethylated CpG site (TG), respectively. *P* < 0.001. **(B)** Association between the ID4 methylation percentage using bisulfite sequencing PCR and methylight. Using linear regression, we obtained a goodness of fit of *r* ^2^ = 0.7168. Using an exponential curve, the goodness of fit was *r* ^2^ = 0.8485. AL, acute leukemia; MDS, myelodysplastic syndrome; NBM, normal bone marrow; TSS, transcriptional start site.

The methylated or unmethylated status of the promoter region of the ID4 gene was determined by PCR amplification of the bisulfite-treated genomic DNA using specific PCR primers (Table 2 and Figure 1).

**Table 2:** **Sequences of the primers and probes**

	**Forward**	**Reverse**
BSP		
ID4 primer	**5′-**GTTTGATTGGTTGGTTATTTTAGAT-**3′**	**5′-**ACCGAAAAAAAAATAACCCAC-**3′**
		**5′-**CACCAAAAAAAAAATAACCCAC-**3′**
Methylight PCR		
MYOD1 primer	**5′-**CCAACTCCAAATCCCCTCTCTAT-**3′**	**5′-**TGATTAATTTAGATTGGGTTTAGAGAAGGA-**3′**
MYOD1 probe	**5′-**TCCCTTCCTATTCCTAAATCCAACCTAAATACCTCC-**3′**	
ID4 primer	**5′-**TCGGAGTTTTCGTTTTCGTT-**3′**	**5′-**CGATACTACTCACAACCGCG-**3′**
ID4 probe	**5′-**AGCGGGTTTCGTTCGGTTCG-**3′**	

ID4, inhibitor of DNA binding 4; BSP, bisulfite sequencing PCR.

The PCR conditions were 95°C for 15 min, followed by 40 cycles of 50 s at 94°C, 45 s at 53°C, and 60 s at 72°C, and a final extension of 72°C for 10 min. The PCR reaction mixture contained 2 μl of bisulfite-modified DNA, 1 μl of 25 mM dNTPs, 2.5 μl of 10× Herman’s buffer, 1.5 μl of 10 pmol/μl of each primer, and 0.5 μl of Hotstar Taq DNA Polymerase (Invitrogen, Carlsbad, CA, USA), for a final volume of 25 μl.

PCR products were analyzed on a 1.5% agarose gel, and then were purified using a QIAquick Gel Extraction Kit (Qiagen) and transformed into *Escherichia coli* cells using the pGEM-T easy vector (Promega). Ten individual clones were selected for vector extraction by QIAprep Spin Mini Prep Kit (Qiagen). Vector DNA samples were sequenced by Invitrogen.

### Methylight

After bisulfite treatment and purification, the promoter of the ID4 gene was evaluated by methylight. The primers and the probe for methylight were located in the BSP-amplified sequences. The primers and the ID4 gene probe had 10 CpG sites. The primers and the internal control gene MYOD1 probe had no CpG sites [37]. Both the ID4 gene and MYOD1 gene probes were labeled with 6-fluorescein amidite (FAM) at the 5′ end and the quencher tetramethylrhodamine (TAMRA) at the 3′ end. All primers and probes were synthesized by Invitrogen. The primers and probes used for PCR are shown in Table 2.

The 20-μl reaction mixture included 2.5 μmol of each primer, 1.25 μmol of probe, 2 μl of bisulfite-converted DNA, and 10 μl of mix buffer (Qiagen). Conditions were as follows: 95°C denaturation for 10 min, followed by 45 cycles at 95°C denaturation for 30 s, and 57°C annealing and extension for 1 min. Methylight PCR reactions were performed in an MX3000p device (Stratagene, La Jolla, CA, USA). To control the accuracy of the methylight reactions, we used DNA from NB4 cell lines previously known to be methylated and DNA from 293 cell lines previously known to be unmethylated for the genes being analyzed.

The methylation levels of the ID4 gene were calculated individually using the relative quantification method. The standard sample was made of bisulfite-treated DNA from the NB4 cell line. A standard curve was produced for ID4 and MYOD1 (as the internal control gene) [37] by a 10-fold dilution series of four different plasmid concentrations. Every run had a standard curve. The positive samples had amplification curves, but negative samples had no amplification curves.

### Statistical analysis

Statistical analysis was performed using SPSS 18.0 for Windows (IBM, Armonk, NY, USA). We used the chi-square test or the Fisher’s exact test to evaluate categorical data. The Mann–Whitney non-parametric test was used for comparisons of continuous data.

The Spearman’s rank correlation was used for correlation of methylation levels and bone marrow blast levels.

Overall survival (OS) was calculated from diagnosis day until the day of death or the last follow-up date (censored on 31 December 2012). OS was analyzed according to the Kaplan-Meier method and the log-rank test. The Cox proportional hazard regression model was used for the adjustment of independent prognostic factors in multivariate survival analysis. Two-tailed *P* values ≤ 0.05 were considered statistically significant.

## Results

### Detection of CpG methylation frequency by BSP

We detected the methylation status of bone marrow samples from one healthy donor, one MDS patient, and one AML patient using BSP. Ten clones from every bone marrow sample were chosen. For the ID4 gene, every clone had 48 CpG sites. The frequency of positive sites in the ID4 gene was 2.08% (10/480) for the normal bone marrow (NBM) sample, 41.46% (199/480) for the MDS patient sample, and 70.00% (336/480) for the MDS-AL patient sample (Figure 1A).

### Comparison of ID4 gene methylation status in NBM, MDS, and MDS-AL by methylight

ID4 methylation positivity rates were different among the NBM (0%), patients with MDS (27%) and patients with MDS-AL (68.18%). ID4 methylation levels were also different among the NBM [0 (0 to 0)], patients with MDS [0.21 (0 to 3.79)], and patients with MDS-AL [0.57 (0 to 1.43)]. Both methylation positivity rates and methylation levels were significantly different between healthy donors, patients with MDS and patients with MDS-AL (*P* < 0.001, *P* < 0.001, Table 3). There was an association between the ID4 methylation percentage using BSP and methylight. Using linear regression, we obtained a goodness of fit of *r*^2^ = 0.7168. Using an exponential curve, the goodness of fit was *r*^2^ = 0.8485 (Figure 1B).

**Table 3:** **ID4 methylation status of NBM, MDS, and AML**

	**NBM**	**MDS**	**MDS-AL**	***P*** **value**
Patients (*N*)	20	100	22	
ID4 methylated	0	27	15	<0.001
ID4 methylated level	0 (0 to 0)	0.21 (0 to 3.79)	0.57 (0 to 1.43)	<0.001

NBM, normal bone marrow; MDS, myelodysplastic syndrome; AL, acute leukemia; ID4, inhibitor of DNA binding 4.

### Detection of ID4 gene methylation status by methylight and correlation with clinical characteristics

**Figure 2:**
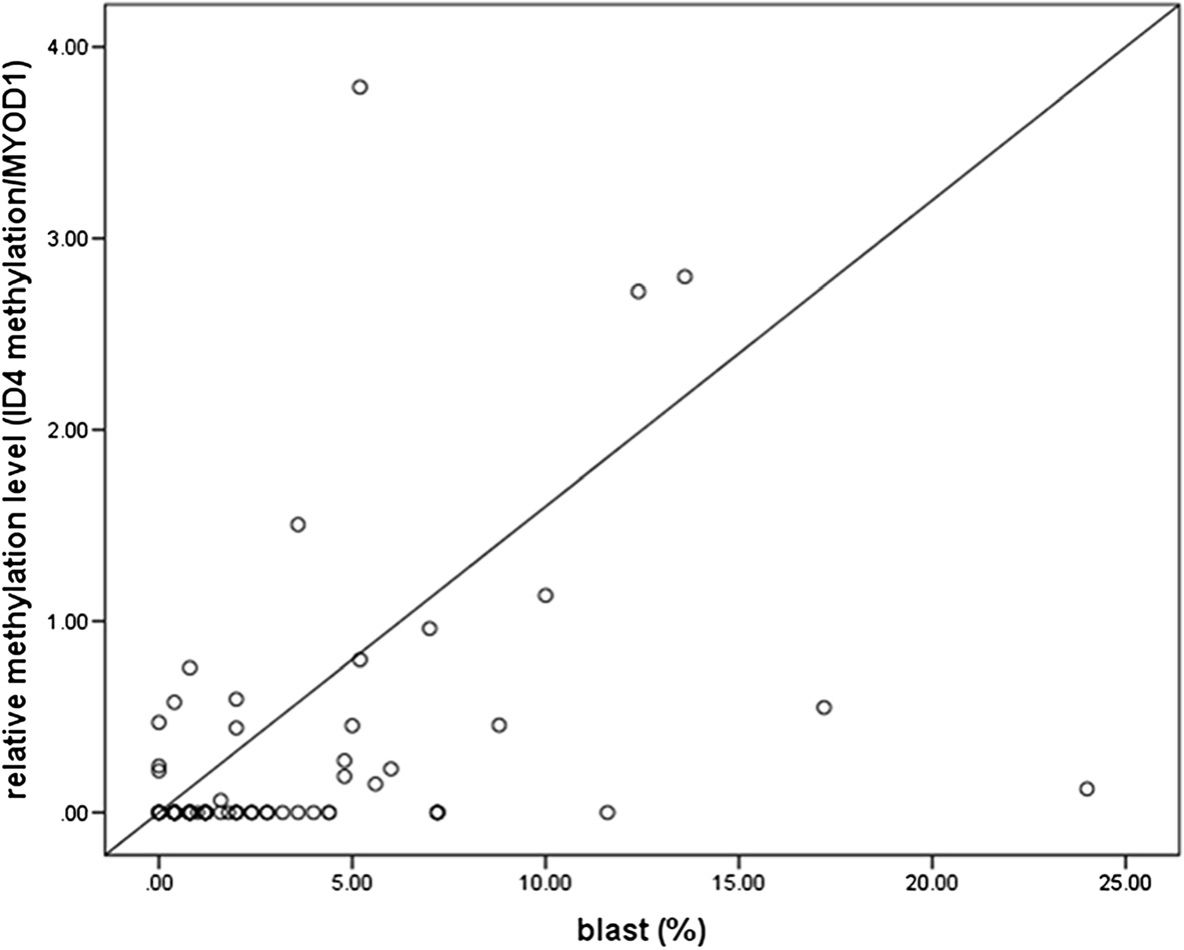
**Methylation level of the ID4 gene according to the percentage of bone marrow blasts.** ID4 gene methylation levels increase with the number of bone marrow blasts (*r* = 0.388, *P* < 0.001). ID4, inhibitor of DNA binding 4.

As summarized in Table 4, 27 patients showed ID4 gene hypermethylation. Patients with ID4 gene methylation had higher levels of white blood cell (WBC) counts and bone marrow blast counts than those without (*P* = 0.036, *P* = 0.001). ID4 methylation levels were correlated with bone marrow blast counts in patients with MDS (*r* = 0.388, *P* < 0.001, Figure 2). There were no significant differences in other clinical features, such as age, sex, initial hemoglobin levels, and platelet counts, between the MDS patients with and without ID4 gene hypermethylation (*P* > 0.05).

**Table 4:** **Correlation between clinical characteristics and ID4 gene methylation**

	**Total**	**Methylated**	**Unmethylated**	***P*** **value**
Patients (N)	100	27	73	
Age (years)	46 (13 to 86)	51 (38 to 83)	44 (13 to 86)	0.100
Sex (male/female)	55/45	14/13	41/32	0.700
WBC (×10^9^/L)	4.12 (0.35 to 25.9)	3.95 (0.352 to 20)	4.18 (1.05 to 25.9)	0.036
Hemoglobin (g/L)	86 (44 to 140)	76 (52 to 114)	89 (44 to 140)	0.088
Platelet (×10^9^/L)	84.19 (1 to 459)	98.43 (12 to 459)	90.16 (1 to 381)	0.656
BM blasts (%)	2.99 (0 to 17.2)	6.1 (0 to 17.2)	1.8 (0 to 11.6)	0.001

BM, bone marrow; WBC, blood cell counts.

### Association between ID4 gene methylation status (by methylight) and MDS subtypes

As summarized in Table 5, those who harbored ID4 gene hypermethylation according to WHO subtypes included four (9.76%) of the 41 patients with RA, four (18.18%) of the 22 patients with refractory cytopenia with multilineage dysplasia (RCMD), one (14.29%) of the 7 patients with refractory anemia with ringed sideroblasts (RARS), eleven (52.38%) of the 21 patients with refractory anemia with excess blasts (RAEB)-1, and seven (77.78%) of the 9 patients with RAEB-2. There was a significant difference in the rates of ID4 gene methylation between the different WHO subtypes (*P* < 0.001). MDS patients with high-risk subtypes (RAEB-1 and RAEB-2) had a significantly higher incidence of ID4 gene methylation (60%) than those with low-risk subtype (RA, RCMD, and RARS, 12.86%, *P* < 0.001). Another risk stratification, the IPSS (which weights the effects of blast quantity, karyotype, and cytopenia), also showed a different methylation positivity rate between the low-risk group (8.33%, 2/24 patients with low and intermediate risk (INT) 1) and the high-risk group (63.64%, 7/11 patients with INT 2 and high) (*P* = 0.002). As summarized in Table 5, ID4 gene methylation levels was used to evaluate the WHO subtypes (*P* < 0.001), WHO risk stratification (*P* < 0.001), and IPSS risk stratification (*P* = 0.007). However, both the ID4 gene methylation positivity rates and methylation levels of the MDS patients with different karyotypes showed no significant differences (*P* = 1.000, *P* = 1.000).

**Table 5:** **Association between ID4 gene methylation status and MDS subtypes**

	**Total**	**Methylation level**	***P*** **value**	**Methylated**	***P*** **value**
Patients (*N*)	100			27	
IPSS karyotype	37		0.360	9	0.298
Good	22	0.14 (0 to 2.72)		4	
Intermediate	9	0.24 (0 to 0.80)		4	
Poor	6	0.04 (0 to 0.24)		1	
BM blast (%)			0.098		<0.001
<5	75	0.47 (0 to 1.51)		10	
5 to 10	13	0.84 (0 to 3.79)		8	
11 to 19	12	1.03 (0 to 2.80)		9	
IPSS cytopenias			0.734		0.787
0/1	22	0.24 (0 to 2.80)		5	
2/3	78	0.20 (0 to 3.79)		22	
IPSS risk group	35		0.007	9	0.002
Low-risk (low/int-1)	24	0.03 (0 to 0.76)		2	
High-risk (Int-2/high)	11	0.43 (0 to 2.72)		7	
WHO subtype	100			27	
Risk			0.000		<0.001
Low risk	70	0.06 (0 to 1.51)		9	
High risk	30	0.54 (0 to 3.79)		18	
Subtype			0.000		<0.001
RA	41	0.04 (0 to 0.59)		4	
RCMD	22	0.06 (0 to 0.76)		4	
RARS	7	0.22 (0 to 1.51)		1	
RAEB-1	21	0.41 (0 to 3.79)		11	
RAEB-2	9	0.8 (0 to 2.8)		7	

RA, refractory anemia; RARS, refractory anemia with ringed sideroblasts; RCMD, refractory cytopenia with multilineage dysplasia; RCMD-RS, refractory cytopenia with multilineage dysplasia and ringed sideroblasts; RAEB, refractory anemia with excess of blasts; IPSS, International Prognostic Scoring System. Good: normal, −Y, del(5q) or del(20) as the sole abnormality; poor: complex (≥3 abnormalities) or chromosome 7 anomalies.

### Prognostic factors analysis

A multivariate Cox survival analysis of age, sex, hemoglobin levels, platelet (PLT) counts, WBC counts, bone marrow blast levels, subtypes by IPSS and ID4 methylation level on OS was performed for the MDS patients (Table 6). Results showed that sex, age, IPSS risk group, and ID4 methylation levels were significant independent risk factors of poor OS in patients with MDS. IPSS risk group was the strongest factor on OS (HR = 7.374).

**Table 6:** **Multivariate analysis of prognostic factors for overall survival**

	***P*** **value**	**HR (95% CI)**
Age (years)	0.007	6.277 (2.417 to 16.300)
Sex (male/female)	<0.001	4.119 (1.466 to 11.578)
Hemoglobin (g/L)	0.731	0.997 (0.983 to 1.012)
WBC (×10^9^/L)	0.738	1.014 (0.935 to 1.100)
Platelet (×10^9^/L)	0.823	1.000 (0.995 to 1.004)
BM blast (%)	0.245	1.041 (0.973 to 1.114)
IPSS risk group	<0.001	7.374 (3.746 to 14.518)
ID4 methylation level	0.011	2.371 (1.221 to 4.602)

BM, bone marrow; WBC, blood cell counts; IPSS, International Prognostic Scoring System; ID4, inhibitor of DNA binding 4, 95% CI, 95% confidence interval.

The estimated median survival time of all patients was 39.0 months. Using the IPSS risk groups, patients with a higher risk had a shorter median survival (51.4 months for the low risk group, 42.1 months for the INT 1 risk group, 35.2 months for the INT 2 risk group, and 22.0 months for the high risk group).

**Figure 3:**
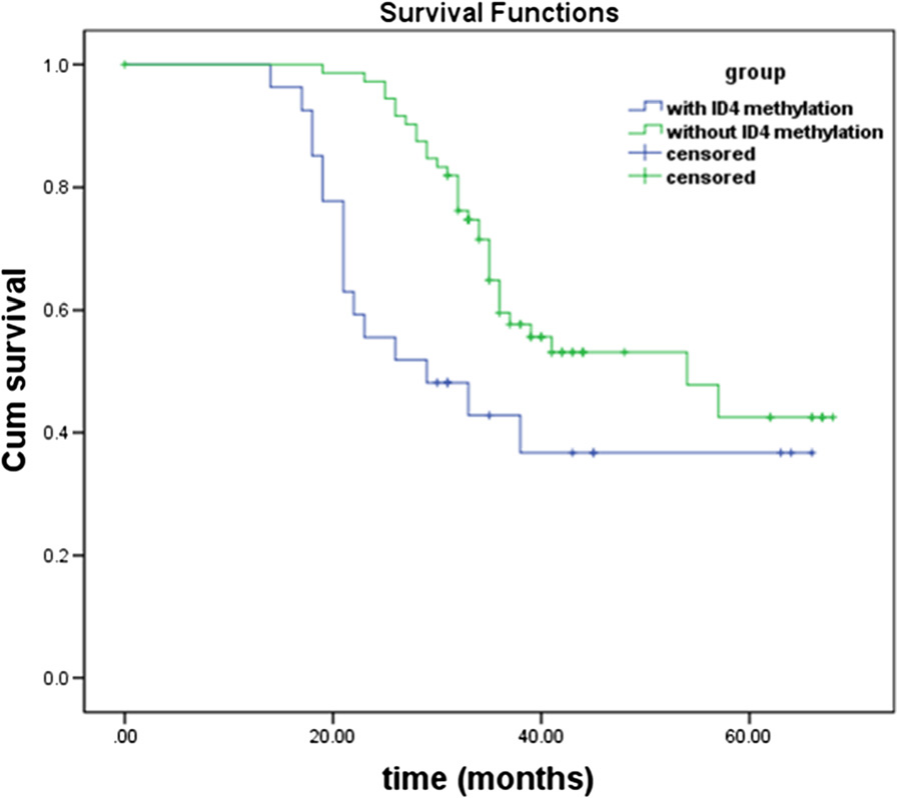
**Overall survival of MDS patient groups for ID4 gene with and without methylation.** Patients with ID4 methylation had a shorter survival than those without ID4 methylation (*P* = 0.008). ID4, inhibitor of DNA binding 4.

ID4 methylation level was an independent factor affecting OS. Patients with MDS showed poor survival with increased ID4 methylation levels (*P* = 0.011, HR = 2.371). According to the multivariate analysis, age and sex were significant predictors of OS (*P* = 0.007, HR = 6.277 and *P* < 0.001, HR = 4.119, respectively), whereas marrow blast levels did not show statistical significance (*P* = 0.245). Patients with ID4 methylation had shorter survival time than those who had unmethylated ID4 (*P* = 0.008, Figure 3). There was a significant difference in the median survival between the methylated ID4 group and the unmethylated group (30.2 and 44.3 months, respectively).

### Sequential analysis of ID4 gene methylation status during MDS evolution

**Figure 4:**
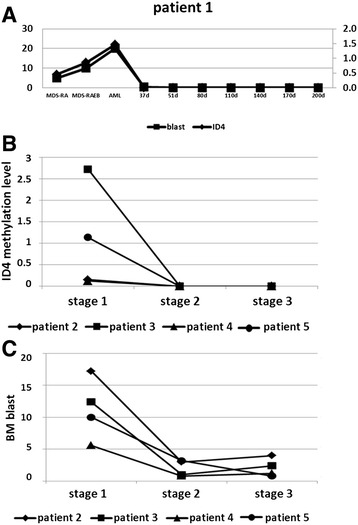
**Relationship between ID4 methylation levels and BM blasts during disease status in five MDS patient samples at different disease stages.** ID4 gene methylation levels increased or decreased with the number of bone marrow blasts. **(A)** ID4 methylation level of one patient during different disease stages MDS-RA, MDS-RAEB, AML, day 37 to day 200 after transplantation. **(B)** ID4 methylation level of four patients in different disease stages. **(C)** BM blasts of four patients in different disease stages. Stage 1: new diagnosis; stage 2: the first period of demethylation treatment for patient 2 and patient 3 and 1 month after transplantation for patient 4 and patient 5; stage 3: the second period of demethylation treatment for patient 2 and patient 3 and 2 months after transplantation for patient 4 and patient 5. AML, acute myeloid leukemia; ID4, inhibitor of DNA binding 4; MDS-RA, MDS with refractory anemia; MDS-RAEB, MDS with refractory anemia with excess blasts.

For one patient, bone marrow samples were available over five stages. With a follow-up time of 20 months, this patient underwent a disease progression from MDS-RA and MDS-RAEB to AL and transplantation. ID4 methylation levels of MDS-RA, MDS-RAEB, AL, and days 37 to 200 after transplantation were 0.45, 0.86, 1.47, and 0, respectively. ID4 gene methylation levels changed with the number of bone marrow blasts. Four additional patients with MDS with sequential samples were in the same situation: ID4 gene hypermethylation reflected disease stage and became negative after transplantation or demethylation treatment, and this was associated with bone marrow blasts (Figure 4).

## Discussion

The aim of this study was to investigate the biological significance and the prognostic relevance of the ID4 gene methylation status and methylation levels in MDS patients. Our results, which used for the first time quantitative real-time methylation-specific PCR (methylight) for ID4 gene methylation detection, indicated that aberrant methylation of the ID4 gene may play an important role in the prognosis of MDS.

Firstly, we detected the methylation of the ID4 gene promoter region CpG sites using BSP. There was a sharp increase in methylation positivity rates from NBM, MDS to AL, which corresponded with the methylight result. As the intermediate stages of AL, MDS had moderate methylation levels of the ID4 gene, intermediate to those for NBM and AL. This is a clear indication that ID4 methylation detection could be used to monitor MDS progression.

In this study, 27 patients showed aberrant ID4 gene methylation, which was present in each WHO subtype of MDS, with increasing amounts in ascending order of RA, RCMD, RARS, RAEB-1, and RAEB-2. Methylation occurred more frequently in both high-risk WHO and IPSS subtypes of MDS. Wang et al. reported that ID4 methylation was present in 35.4% of adult patients newly diagnosed with MDS and occurred more frequently in advanced-stage MDS by methylation-specific PCR (MSP) [35]. These discrepancies may be the result of the different methods used. Besides the methylation positivity rate, methylation levels were also used to evaluate the relationship with the WHO subtypes and IPSS. ID4 gene hypermethylation occurred in more advanced stage of MDS samples, as well as with higher methylation levels. As an epigenetic abnormality, high ID4 gene hypermethylation may increase the risk of disease progression in patients with MDS patients.

Many studies have reported that more bone marrow blasts in patients with MDS provide more opportunities for leukemia transplantation [38]. Previously, it was shown that both bone marrow blasts and ID4 methylation were independent factors for leukemia-free survival (LFS) estimation in MDS patients [35]. In our study, not only ID4 methylation levels had high correlation with bone marrow blasts, but there was also a significant difference between the ID4 methylated and unmethylated groups, in terms of bone marrow blasts. This hinted that ID4 methylation levels may be a substitute for the degree of dysplastic cells for interpreting pathogenetic conditions. After treatment by either transplantation or demethylation, ID4 gene methylation in all sequential samples was negative, corresponding to decreased bone marrow blasts. To some extent, ID4 gene methylation may be a potential new marker for minimal residual disease (MRD) detection. In addition, the detection of ID4 gene methylation could offer some guidance to MDS treatment stratification.

In our multivariate analysis, IPSS risk groups was an independent factor that affected the OS of patients with MDS. This finding was consistent with their clinical state. Our investigations indicated that the ID4 methylation levels were higher in advanced-stage MDS, indicating that ID4 methylation might be a molecular event playing an important role in the progression of MDS. In our study, ID4 methylation levels were considered to affect the OS of MDS patients. Meanwhile, patients with ID4 methylation had a shorter survival time. These results hinted that ID4 methylation levels may be an independent prognostic factor for overall survival.

Our results revealed that while there was no significant correlation between ID4 gene methylation and genetic abnormalities, taken together they may offer greater potential as biomarkers for MDS. As previously reported, the occurrence and progression of cancers includes both genetic and epigenetic alterations [39,40], and for epigenetic alterations, ID4 gene methylation could be used for auxiliary diagnosis and prognosis of MDS patients without significant genetic alterations. Combining these two mechanisms could cover more scope for disease monitoring, treatment effect evaluation and MRD detection.

The present study investigated the status and levels of ID4 gene methylation in seven patients with MDS at different stages of MDS to directly interpret the close correlation of disease evolution with ID4 gene methylation for the first time. In terms of disease progression, ID4 gene methylation status of one sequential MDS patient sharply increased from MDS-RA and MDS-RAEB to AML. After transplantation, ID4 methylation was negative, supporting the role of ID4 gene methylation in MRD progression.

It is noteworthy that drugs with demethylating activity are increasingly used and studied in hematological malignancies [41-43]. One could speculate that the presence of increased DNA methylation levels in MDS in clinical remission might provide a rationale for the use of these drugs in affected patients. Further clinical studies are necessary to clarify this possibility and to define the accuracy of methylation MRD estimation.

## Conclusions

Our findings provide evidence that aberrant DNA methylation is present in MDS patients and can potentially be used for prognosis. These findings may offer the possibility of prognostic estimation for many patients who currently lack adequate markers.

## Abbreviations

AL: acute leukemia
AML: acute myeloid leukemia
ATCC: American type culture collection
BSP: bisulfite sequencing PCR
FAM: 6-fluorescein amidite
ID4: inhibitor of DNA binding 4
INT: Intermediate risk
IPSS: International Prognostic Scoring System
MDS: myelodysplatic syndrome
NBM: normal bone marrow
OS: overall survival
PCR: polymerase chain reaction
PLT: platelet
RA: refractory anemia
RAEB: refractory anemia with excess blasts
RARS: refractory anemia with ringed sideroblasts
RCMD: refractory cytopenia with multilineage dysplasia
TAMRA: tetramethylrhodamine
TSG: tumor suppressor gene
WBC: white blood cells
WHO: World Health Organization
